# Instance-Level Maturity Recognition of *Pleurotus citrinopileatus* via Ordinal Learning and Morphology-Guided Feature Fusion

**DOI:** 10.3390/jof12100737

**Published:** 2026-10-01

**Authors:** Qingfeng Wei, Yaming Zheng, Rupeng Luan, Yang Lu, Qian Zhang, Ruifang Zhao, Chenzhong Cao, Jun Yu, Changshou Luo

**Affiliations:** 1 Institute of Data Science and Agricultural Economics, Beijing Academy of Agriculture and Forestry Sciences, Beijing 100097, China; weiqingfeng@baafs.net.cn (Q.W.);; 2 Beijing Engineering Research Center of Rural Remote Information Services, Beijing 100097, China; 3 Key Laboratory of Intelligent Seedling Technology Innovation, Ministry of Agriculture and Rural Affairs, Beijing 100097, China; 4 Beijing Key Laboratory of Crop Molecular Design and Intelligent Breeding, Beijing 100097, China

**Keywords:** *Pleurotus citrinopileatus*, maturity recognition, instance detection, ordinal classification, dual-view learning, morphology-guided feature fusion, computer vision

## Abstract

Accurate maturity recognition of *Pleurotus citrinopileatus* fruiting bodies is important for growth monitoring and harvest assistance in controlled cultivation. However, multiple developmental stages often coexist within one image, and near-mature fruiting bodies share visual characteristics with both adjacent stages. We developed a two-stage RGB-based framework for instance-level maturity recognition in complex cultivation scenes. After fruiting-body localization, each detected instance was represented by paired local and context views and classified using a DINOv3 ViT-S/16-based ordinal model. The task-specific methodological contribution is a morphology-guided fusion module that incorporates automatically extracted geometric, color, intensity, and edge descriptors into the dual-view visual representation. Object-level matching was used to quantify how detection and classification errors affected the complete pipeline, and temperature scaling was applied for probability calibration. Among five candidate detectors, RF-DETR-Small achieved validation mAP50 and mAP50–95 values of 0.9747 and 0.8813, respectively. On the independent ROI test set, the final classifier achieved an accuracy of 0.8779, a macro F1 of 0.7950, a near-mature F1 of 0.5546, and a QWK of 0.9068. Temperature scaling reduced ECE from 0.1094 to 0.0564 without changing the predicted labels. On 370 original cultivation images containing 475 ground-truth instances, the unfiltered pipeline achieved an object-level macro F1 of 0.7072 and a near-mature F1 of 0.4672. Candidate-box filtering increased these scores to 0.7305 and 0.4885, respectively, but increased the number of missed instances from 14 to 23. These results demonstrate the feasibility of non-destructive instance-level maturity monitoring while identifying near-mature discrimination and candidate-box control as priorities for practical application.

## 1. Introduction

Images directly capture the color, morphology, and developmental status of fruiting bodies and are increasingly used for mushroom growth monitoring, quality assessment, and harvest assistance [1,2]. Existing studies, however, have focused mainly on object detection, sorting, phenotypic measurement, or visual perception for harvesting equipment, whereas fine-grained recognition of the maturity state of individual fruiting bodies in complex cultivation scenes remains less explored [3]. In *P. citrinopileatus* production, multiple fruiting bodies at different developmental stages commonly coexist within a single field of view, making whole-image maturity assessment poorly suited to representing individual variation. Reliable instance-level maturity recognition from conventional RGB images is therefore needed to represent this variation and support harvest decisions [4].

Previous studies have used YOLOv5 and Detectron2 to monitor oyster mushroom growth and estimate harvest dates [5]. Mask R-CNN has been used to integrate instance segmentation, localization, temporal tracking, and area estimation [6], while a lightweight detector combined with a growth-curve model has been applied to growth-stage recognition [7]. More recent studies using YOLOv8-seg and YOLOv11-seg have demonstrated the value of instance segmentation for fruiting-body measurement and mushroom phenotyping [8]. Collectively, these studies have advanced mushroom localization, phenotypic measurement, and coarse growth-stage analysis. However, fine-grained recognition of individual *P. citrinopileatus* fruiting bodies, particularly discrimination among adjacent maturity stages, remains underexplored.

A first challenge in instance-level maturity recognition of *P. citrinopileatus* is how to organize target and background information in complex cultivation scenes. Raw cultivation images usually contain multiple fruiting bodies together with cultivation bags, substrate, support structures, and neighboring individuals. Whole-image input may introduce background information unrelated to the target, whereas an overly tight crop may exclude useful information about neighboring fruiting bodies and occlusion. A target-centered representation that retains limited local context may therefore reduce irrelevant background interference while preserving informative contextual cues [9].

Growth and maturation of *P. citrinopileatus* fruiting bodies form a continuous process, and the immature, near-mature, and mature stages have an inherent order without sharply defined visual boundaries. Near-mature fruiting bodies are particularly difficult to distinguish because cap size, expansion, margin curvature, color, and overall morphology often overlap with characteristics of both adjacent classes. Treating the three stages as mutually independent categories does not explicitly exploit this ordered structure. Ordinal modeling can incorporate inter-class ranking information and reduce implausible errors between distant maturity stages [10]. Deep visual features capture color, texture, and local structure, whereas explicit descriptors of region proportions, edge characteristics, and color statistics may provide complementary information near stage boundaries. Combining ordinal modeling with automatically extracted morphology descriptors therefore provides a biologically relevant basis for distinguishing adjacent maturity stages.

Instance-level maturity recognition in practical settings is also affected by errors in object detection. Complex backgrounds, occlusion, low illumination, and changes in target scale can produce missed detections, false positives, and localization errors [11]. These errors may prevent true fruiting bodies from entering the classifier, introduce non-target regions, or alter the visual and morphological information extracted from candidate boxes. Consequently, classification performance based only on manually annotated instance crops does not fully represent performance on raw cultivation images. An end-to-end protocol integrating detection, cropping, maturity classification, and object-level matching is therefore required to distinguish localization errors from classification errors. Calibration of prediction confidence is also relevant when model outputs are used to support management decisions or manual review.

Accordingly, this study aimed to develop and evaluate a two-stage framework for instance-level maturity recognition of *P. citrinopileatus* in complex cultivation scenes. We hypothesized that combining target-centered local and contextual information with ordinal stage relationships and explicit morphology would improve discrimination among adjacent maturity stages, particularly the near-mature stage. Candidate detectors were first compared to identify a suitable model for localizing individual fruiting bodies. Each localized instance was subsequently classified using a DINOv3 ViT-S/16-based dual-view ordinal model with morphology-guided feature fusion. Temperature scaling was applied for post-hoc probability calibration, and performance was assessed using both manually defined instance crops and an end-to-end object-level evaluation. The principal contribution lies in the biologically informed integration of instance localization, target-context representation, ordinal modeling, and morphology-guided fusion within a unified framework for individual fruiting-body assessment. Within this framework, morphology-guided fusion constitutes the task-specific methodological design, while object-level matching enables the effects of detection and classification errors to be evaluated separately.

## 2. Materials and Methods

### 2.1. Overall Framework

A two-stage visual analysis pipeline was developed for instance-level maturity recognition of *Pleurotus citrinopileatus* under controlled cultivation (Figure 1). Starting from raw cultivation images, separate datasets were constructed for instance detection and maturity classification. Candidate detectors were trained and evaluated using manually annotated bounding boxes as ground truth, and model selection was based exclusively on validation-set performance. For ROI maturity classification, manually annotated instance boxes were used to generate paired local and context views; backbone selection, model configuration, and related hyperparameters were determined on the validation set. After the model and hyperparameters were fixed, the independent test set was reserved for final performance reporting. In the end-to-end experiment, raw cultivation images were provided directly to the final detector, which automatically generated instance boxes for dual-view cropping, maturity classification, and object-level evaluation. DINOv3 ViT-S/16 served as the visual backbone of the classifier, together with ordinal maturity modeling and morphology-guided feature fusion. Temperature scaling was applied to the final class probabilities. This design separates standalone detection and classification evaluation from end-to-end system evaluation, enabling localization errors, classification errors, and the effects of candidate-box filtering to be analyzed independently.

### 2.2. Experimental Materials, Image Annotation, and Dataset Construction

The experiment was conducted from 11 to 22 January 2025 at the container-type edible-mushroom cultivation facility (12.192 m × 2.438 m × 2.896 m in length, width and height) of Hongze Jiayuan Planting Cooperative in Fangshan District, Beijing, China (Figure 2). Image collection followed a single cultivation batch through one fruiting cycle, allowing all three maturity stages to be recorded within the same production setting. The 11-day observation period extended from the appearance of identifiable immature fruiting bodies to the mature harvest stage. *Pleurotus citrinopileatus* fruiting bodies were grown under controlled conditions and photographed with Nikon D7100 and Nikon D3100 (Nikon Corporation, Tokyo, Japan) digital cameras. Images were saved in JPEG format at original resolutions of 6000 × 4000 pixels for the Nikon D7100 and 4608 × 3072 pixels for the Nikon D3100. The cameras were positioned approximately 0.5–1.0 m from the fruiting bodies. The two camera models were used to introduce moderate variation in sensor resolution and image characteristics and to reduce dependence on a single imaging device. The study was not designed to compare camera performance. Images acquired with both cameras followed the same annotation, dataset-partitioning, cropping, resizing, and normalization procedures. Images were acquired under the existing artificial lighting in the cultivation facility without flash or additional dedicated illumination. Automatic exposure and white balance were used, with ISO sensitivity generally ranging from 200 to 800, shutter speed from approximately 1/30 to 1/125 s, and aperture from f/3.5 to f/5.6. No additional color correction or geometric calibration was applied before annotation. Before model input, the dual-view crops were resized to 320 × 320 pixels and normalized as described in Section 2.4. The images covered variation in maturity stage, fruiting-body size, occlusion, and cultivation background and were used to construct the instance-level maturity dataset.

Image annotation was performed independently by three experts with professional experience in mushroom cultivation and maturity assessment. Before formal annotation, the experts jointly established annotation guidelines covering instance inclusion, bounding-box placement, and the morphological criteria for each maturity class. Annotation was conducted at two levels: instance localization and maturity classification. Each visible *P. citrinopileatus* fruiting body was enclosed within a bounding box. For maturity classification, each annotated instance was assigned to one of three ordered classes: immature, near-mature, or mature. Immature fruiting bodies had incompletely expanded caps, distinctly incurved margins, and a compact overall form. Near-mature fruiting bodies showed partial cap expansion and initial margin flattening, while the margins remained slightly incurved. Mature fruiting bodies had broadly expanded caps with flat or slightly upturned margins. Cap color, fruiting-body size, and overall form were used as supporting cues when cap expansion and margin morphology alone did not permit a clear assignment. The near-mature class represented the transition between the immature and mature stages and could therefore exhibit characteristics of both adjacent classes. A formal inter-annotator agreement coefficient was not calculated. Differences in bounding-box placement or maturity assignment were reviewed jointly by the three experts and resolved by consensus.

Dataset partitioning was performed at the original-image level to prevent information leakage among subsets. All instances from the same source image, together with their derived local and context crops, were assigned to a single subset. The instance-detection and maturity-classification datasets therefore shared the same training, validation, and test split.

The classification dataset comprised 3158 paired dual-view samples, including 2208 training instances, 475 validation instances, and 475 test instances. Each sample contained a local view and a context view generated from the same annotated instance box and assigned the same maturity label. The training set was used for model fitting and estimation of morphology-feature standardization statistics. The validation set was used for model selection, hyperparameter tuning, post-processing threshold search, and temperature estimation. The test set was reserved for final ROI classification after all model and parameter choices had been fixed.

The maturity-class distribution across the training, validation, and test subsets is summarized in Supplementary Table S1. The near-mature class contained 352 samples, representing 11.1% of the classification dataset, including 53 samples in the test set. The three subsets had similar class proportions, although near-mature samples were less represented than immature and mature samples.

The end-to-end test set comprised 370 original cultivation images containing 475 ground-truth instances. Manual bounding boxes were not used as model inputs at this stage. Candidate boxes were generated by the final detector and subsequently used for dual-view cropping, maturity prediction, and object-level matching. Manual annotations were used only as ground truth for end-to-end evaluation.

### 2.3. Instance Detection Model and Model Screening

Instance detection was used to localize individual *P. citrinopileatus* fruiting bodies in raw cultivation images and served as the input stage for subsequent instance-level maturity classification. Because end-to-end performance depends directly on the quality of detected boxes, the detector must provide both high coverage of true instances and effective control of false positives, thereby reducing the number of background regions, cultivation bags, or other non-target structures passed to the classifier.

RF-DETR-Small, YOLO11s, YOLO26s, Faster R-CNN R50-FPN V2, and RT-DETR-L were compared using the same original-image-level data split [12,13,14,15]. All candidate detectors used manually annotated bounding boxes as ground truth, and model screening and related threshold selection were performed on the validation set using predefined detection metrics. The test set did not participate in detector selection and was reserved for evaluation after the final detector had been fixed. Based on validation performance, RF-DETR-Small was used for subsequent end-to-end instance localization. Let the original cultivation image be I, and let the detector $D_{\theta }$ output a set of candidate instance boxes and corresponding confidence scores:

(1) $$B_{p}=D_{\theta }(I)={\left\{\left(b_{p}^{k},s_{p}^{k}\right)\right\}}_{k=1}^{K}$$

where $b_{p}^{k}$ denotes the kth predicted box, $s_{p}^{k}$ is its detection confidence, and K is the number of predicted instances. In standalone ROI classification, local and context crops were generated from manually annotated instance boxes. In end-to-end evaluation, the detector-generated candidate boxes were first subjected to the predefined confidence screening and post-processing steps and were then used for dual-view cropping and maturity classification.

In end-to-end evaluation, predicted and ground-truth boxes were matched using intersection over union (IoU). For a predicted box $b_{p}$ and a ground-truth box $b_{g}$, IoU was defined as:

(2) $$IoU\left(b_{p},b_{g}\right)=\frac{\left|b_{p}\cap b_{g}\right|}{\left|b_{p}\cup b_{g}\right|}$$

An IoU threshold of 0.50 was used for spatial matching in end-to-end evaluation. Within each original image, all prediction-ground-truth pairs satisfying this threshold were sorted by IoU in descending order and greedily matched one-to-one; each predicted box and each ground-truth box could participate in at most one match. Successfully matched predictions were retained for joint maturity evaluation. A predicted box that did not match any ground-truth box was counted as a false-positive instance, whereas a ground-truth instance not matched by any prediction was counted as a missed instance. When multiple predictions overlapped the same ground-truth instance, only the first valid one-to-one match was retained; remaining duplicate predictions were counted as false-positive instances.

### 2.4. Dual-View Crop Generation and Preprocessing

To preserve both fruiting-body morphology and a limited amount of local cultivation context, two crops were generated from each instance box: a local view and a context view [16]. In standalone ROI classification, the instance box was defined by manual annotation, whereas in end-to-end evaluation it was generated by the final detector. The local and context views used expansion factors of 1.0 and 1.4, respectively. Crops were clipped to the original image boundaries, resized to 320 × 320 pixels, and maintained as one-to-one pairs throughout training, validation, and testing.

During training, the same augmentation policy was applied to both views, including random resized cropping, horizontal flipping, mild color jitter, and small-angle rotation. Validation and test images were only resized, converted to tensors, and normalized using the ImageNet mean and standard deviation. A fixed random seed was used for all experiments.

The local view retained target-specific cues such as cap color, margin expansion, and fruiting-body morphology, whereas the context view captured neighboring fruiting bodies, occlusion relationships, and the local cultivation background.

### 2.5. Dual-View Ordinal Maturity Classification Model

DINOv3 ViT-S/16 was used as the visual backbone for maturity classification to extract fine-grained information on morphology, color, edge texture, and local structure from each fruiting-body image [17]. The input consisted of the paired local and context views for the same instance. Both views were encoded by the shared encoder $E_{\theta }$ to obtain:

(3) $$f_{l}=E_{\theta }(x_{l})$$

(4) $$f_{c}=E_{\theta }(x_{c})$$

where $f_{l}$ is the local target feature and $f_{c}$ is the context feature.

The two feature representations were then integrated by a local-context fusion module. The features $f_{l}$ and $f_{c}$ were concatenated and passed through a gating function to generate the fusion weight:

(5) $$\alpha =\sigma \left(W_{\alpha }[f_{l};f_{c}]+b_{\alpha }\right)$$

where $\left[f_{l};f_{c}\right]$ denotes feature concatenation, σ(⋅) is the sigmoid function, and α is the local-context fusion weight. The fused visual representation was defined as:

(6) $$f_{v}=\alpha f_{l}+(1-\alpha )f_{c}$$

The fusion module allows the model to adjust the contribution of the local and context views on a per-sample basis. When target morphology is clearly visible, the local view provides the dominant discriminative information. When occlusion, incomplete boundaries, or insufficient local detail are present, the context view contributes additional environmental and spatial-relation cues.

Because the immature, near-mature, and mature stages have an inherent order, maturity was modeled using ordinal learning. The network first predicted a continuous maturity score from the fused feature representation:

(7) $$z=w_{z}^{T}f_{v}+b_{z}$$

Two learnable ordered thresholds, $\tau_{1}$ and $\tau_{2}$, were then used to map the continuous score to three maturity probabilities, with $\tau_{1}<\tau_{2}$. The class probabilities were defined as:

(8) $$P(y=0|x)=1-\sigma (z-\tau_{1})$$

(9) $$P(y=1|x)=\sigma (z-\tau_{1})-\sigma (z-\tau_{2})$$

(10) $$P(y=2|x)=\sigma (z-\tau_{2})$$

where y=0, y=1 and y=2 correspond to the immature, near-mature, and mature classes, respectively. Unlike conventional classification that treats the three classes as independent, this formulation preserves the ordered maturity structure during prediction and imposes different optimization constraints on adjacent-stage confusion and large-rank errors.

The classification objective combined an ordinal probability loss, an adjacent-rank constraint, boundary-sample weighting, and an uncertainty regularizer. The overall loss was defined as:

(11) $$L=L_{ord}+\lambda_{r}L_{rank}+\lambda_{b}L_{boundary}+\lambda_{u}L_{uncertainty}$$

where $L_{ord}$ is the ordinal probability loss; $L_{rank}$ constrains consistency between maturity scores and class order; $L_{boundary}$ increases the training weight of samples near stage boundaries; and $L_{uncertainty}$ discourages overconfident probability outputs. The coefficients $\lambda_{r}$, $\lambda_{b}$ and $\lambda_{u}$ are the weights of the corresponding loss terms.

### 2.6. Morphology-Guided Feature Fusion

Near-mature samples lie between the immature and mature stages, and their cap expansion, margin morphology, color, and size may overlap with both adjacent classes [18]. To complement morphological information that may not be represented explicitly by deep visual features, morphology-guided feature fusion was incorporated into the final classifier.

Morphology descriptors were extracted automatically from each instance box and its paired local and context crops, without additional manual segmentation annotations. Manual boxes were used for standalone ROI classification, whereas detector-generated boxes were used during end-to-end inference. For each crop, a foreground mask was generated using an Otsu-derived grayscale threshold combined with HSV-based warm-color and saturation criteria.

The resulting morphology vector contained 72 descriptors organized at three levels. Eight box-level descriptors recorded normalized width, height, area, horizontal and vertical center coordinates, aspect ratio, logarithmic aspect ratio, and square ratio. Thirty descriptors were then calculated separately from the foreground regions of the local and context crops. Nine geometric descriptors comprised foreground area fraction, normalized foreground-box width and height, aspect ratio, region extent, normalized center coordinates, scale-normalized perimeter, and compactness. Seventeen color and intensity descriptors were also calculated. These comprised the mean and standard deviation of each RGB channel and hue, together with the mean, standard deviation, and interquartile range of saturation, value, and grayscale intensity. Four further descriptors measured mean grayscale gradient, edge density, yellow index, and mean red-green difference. The yellow index was defined as the foreground mean of (R + G)/2 − B, and the red-green difference as the foreground mean of R − G. Finally, four cross-view descriptors recorded the local-to-context foreground-area ratio and the differences in mean value, mean saturation, and yellow index between the two views.

All 72 descriptors were standardized using feature-wise means and standard deviations estimated from the training set. The standardized vector was projected into the visual-feature space through a lightweight multilayer perceptron and combined with the dual-view representation by residual weighted fusion. The fusion weight and associated hyperparameters were selected on the validation set. The selected configuration was then fixed for the final ROI classification and end-to-end evaluations.

The auxiliary morphology classification branch was evaluated only as a model variant in the component-comparison and ablation experiments. Because this branch was structurally coupled with other components in some configurations, the experiments were interpreted as comparisons between complete model configurations. Validation performance was used to determine the final classifier configuration.

### 2.7. Model Training and Evaluation

The classification models were trained for up to 100 epochs using AdamW with a batch size of 16, an initial learning rate of $3\times 10^{-5}$, and a weight decay of 0.05. The learning rate was increased linearly during the first five epochs and then reduced according to a cosine schedule. Both local and context images were resized to 320 × 320 pixels. Early stopping was applied when the validation macro F1 did not improve for 15 consecutive epochs. All experiments used a fixed random seed of 42 and mixed-precision training. Backbone screening, maturity-modeling experiments, morphology-fusion experiments, and component comparisons used the same original-image-level data split and main training protocol. The final morphology-fusion classifier was initialized from the previously trained dual-view ordinal model and fine-tuned under the same settings. Across model configurations, only the components specified for each comparison were changed.

Model selection and hyperparameter tuning were performed exclusively on the validation set, and the independent test set was used only after all settings had been fixed. Detector selection was based primarily on validation mAP50–95, with precision and recall as supporting metrics. ROI-classifier selection used validation macro F1 as the primary metric, with quadratic weighted kappa (QWK), mean absolute error (MAE), and near-mature F1 as complementary measures. The morphology-fusion weight, calibration parameter, selective-prediction threshold, and candidate-box filtering thresholds were also determined on the validation set. The final classifier comprised DINOv3 ViT-S/16, local-context dual-view ordinal learning, morphology input, and morphology-guided feature fusion without a morphology auxiliary loss.

After training, temperature scaling was applied for post-hoc probability calibration. The temperature was estimated on the validation set and then fixed for the independent test set and end-to-end evaluation.

Candidate-box filtering was evaluated as an optional post-processing step for suppressing false-positive instances. Candidate reliability was assessed using detection confidence, classification confidence, and maturity-prediction consistency, with all rules and thresholds fixed on the validation set. Its effect on false positives was evaluated together with the corresponding change in recall.

Detection performance was evaluated using mAP50, mAP50–95, precision, and recall. Classification performance was assessed using accuracy, balanced accuracy, macro F1, near-mature F1, QWK, MAE, severe error rate, and expected calibration error (ECE). Matched, missed, and false-positive instances were additionally counted in the end-to-end evaluation, and error patterns were examined using confusion matrices and class-specific metrics.

End-to-end performance was reported at both the object and matched-instance levels. Predicted and ground-truth boxes were matched one-to-one at IoU ≥ 0.50 according to the procedure described in Section 2.3. At the object level, unmatched predictions were counted as false positives and unmatched ground-truth instances as false negatives. A matched but misclassified instance contributed a false negative to its true class and a false positive to its predicted class. Object-level macro F1 was the mean of the three class-specific F1 scores, whereas object-level near-mature F1 was the F1 score for the near-mature class. Matched-instance metrics were calculated only for successfully matched pairs, thereby separating maturity-classification performance from the effects of missed and unmatched detections.

### 2.8. Use of Generative AI and AI-Assisted Tools

During the preparation of this manuscript, Figure 3 was prepared entirely by the authors without AI-assisted generation or modification. The authors used ChatGPT with GPT-5.5 (OpenAI) to assist with English translation and language polishing. GPT-5.5 was also used through the Nature Skill workflow to assist with the visual refinement and layout optimization of Figure 4 and Figure 5. AI assistance for Figure 4 and Figure 5 was limited to visual presentation and layout and did not alter the underlying data, numerical results, labels, or scientific content. GPT-5.5 was not used to generate or modify experimental data, perform statistical analyses, train or select models, or draw scientific conclusions. All AI-assisted outputs were reviewed and edited by the authors, who take full responsibility for the accuracy and integrity of the final manuscript.

## 3. Results

### 3.1. Instance Detection Performance

Five candidate detectors were compared on the validation set (Table 1). YOLO11s achieved the highest mAP50 (0.9751), whereas RF-DETR-Small achieved the highest mAP50-95 (0.8813), with a precision of 0.9371 and a recall of 0.9411. Faster R-CNN R50-FPN V2 obtained the highest recall (0.9558), but its precision and mAP50-95 were both lower than those of RF-DETR-Small. Because subsequent maturity classification relies on instance crops generated from detected boxes, mAP50-95 was used as the primary detector-selection metric, with precision and recall considered to assess candidate-box quality and instance coverage. Based on validation-set performance, RF-DETR-Small was selected as the instance detector for the subsequent end-to-end maturity-recognition pipeline.

### 3.2. Backbone and Maturity-Modeling Comparison

During backbone screening, five backbone networks were trained using the same data split, maturity labels, and cross-entropy loss and were compared on the validation set (Table 2). ConvNeXtV2-Tiny achieved the highest accuracy (0.8592), with a QWK of 0.8738 and an MAE of 0.1681. DINOv3 ViT-S/16 achieved a slightly lower accuracy (0.8508) but the highest balanced accuracy (0.7441), macro F1 (0.7439), and near-mature F1 (0.4466) among the evaluated backbones. Given the emphasis on overall performance under class imbalance and on recognition of the near-mature class, DINOv3 ViT-S/16 was selected as the backbone for subsequent maturity modeling.

After selecting the backbone, different maturity-modeling strategies were compared using DINOv3 ViT-S/16 (Table 3). Among the single-view approaches, CORN achieved the highest macro F1 (0.7517) and near-mature F1 (0.4685), indicating that this ordinal formulation was well suited to the current maturity-classification setting. CORAL achieved an accuracy of 0.8611 and a QWK of 0.8770, but its near-mature F1 was 0, indicating that it failed to identify the intermediate transition class effectively under the present experimental conditions.

The dual-view ordinal baseline, which combined local-context inputs, ordinal maturity modeling, and boundary weighting, achieved an accuracy of 0.8716, a balanced accuracy of 0.7996, a macro F1 of 0.7873, a near-mature F1 of 0.5333, and a QWK of 0.9035 on the validation set, with an MAE of 0.1389. This configuration provided a favorable balance among overall classification performance, near-mature recognition, and ordinal consistency and was therefore used as the baseline for subsequent morphology-fusion experiments.

### 3.3. Ablation Study of the Morphology-Guided Ordinal Classifier

After the DINOv3 ViT-S/16-based local-context dual-view ordinal classification framework had been established, different configurations of morphology input, feature fusion, and related training components were evaluated on the validation set (Table 4). All models used the same original-image-level data split, random seed, backbone, and main training settings, and the comparison was used to determine the final classifier configuration.

The morphology auxiliary loss was first evaluated while the remaining settings were held constant. Removing this auxiliary loss increased accuracy, macro F1, near-mature F1, and QWK from 0.8653, 0.7661, 0.4771, and 0.8951 to 0.8737, 0.7871, 0.5299, and 0.9052, respectively, while reducing MAE from 0.1495 to 0.1368. Thus, additional morphology-based auxiliary supervision did not improve performance under the current training protocol. The final classifier therefore retained morphology input and feature fusion but excluded the morphology auxiliary loss.

The contribution of the morphology pathway was further examined by comparing the final configuration with the variant in which morphology input was removed. Retaining morphology input and morphology feature fusion without the auxiliary loss increased accuracy, macro F1, and near-mature F1 by 1.05, 2.05, and 4.71 percentage points, respectively, and reduced MAE from 0.1474 to 0.1368. Because removal of morphology input also disabled the corresponding fusion pathway, this comparison reflects the complementary value of the complete morphology-information pathway rather than the isolated effect of either the input or fusion component alone.

The remaining variants showed different trade-offs between overall classification and near-mature recognition. Removing the local-context gate yielded the highest near-mature F1 (0.5484), but QWK decreased to 0.8922 and MAE increased to 0.1474. In comparison, the configuration without the morphology auxiliary loss achieved a higher QWK (0.9052) and a lower MAE (0.1368) while maintaining competitive macro F1 and near-mature F1. The variant without the ranking loss achieved a macro F1 of 0.7683, a near-mature F1 of 0.4821, and a QWK of 0.8980, indicating that different component combinations affected categorical discrimination and ordinal consistency differently.

Based on the validation-set component comparison, the final classifier used DINOv3 ViT-S/16 as the visual backbone together with local-context dual-view ordinal learning and morphology-guided feature fusion, while excluding the morphology auxiliary loss. This configuration provided a favorable balance among overall classification performance, near-mature recognition, and ordinal consistency and was used for the subsequent independent test-set and end-to-end evaluations.

### 3.4. Final Classification Performance and Probability Calibration

After model architecture and related parameters were fixed using the validation set, the final morphology-guided ordinal classifier was evaluated on the independent test set, and its probability calibration was further examined (Table 5). Before calibration, the model achieved an accuracy of 0.8779, a balanced accuracy of 0.8053, a macro F1 of 0.7950, and a near-mature F1 of 0.5546; QWK and MAE were 0.9068 and 0.1326, respectively.

The temperature parameter was estimated on the validation set and then fixed for application to the test set. Because temperature scaling did not change the predicted class labels, accuracy, balanced accuracy, macro F1, near-mature F1, QWK, and MAE remained unchanged, whereas ECE decreased from 0.1094 to 0.0564. This reduction indicates improved agreement between predicted confidence and empirical correctness after calibration.

A near-aware selective decision rule was also examined for handling low-confidence samples. At a coverage of 0.9074, retained-sample accuracy increased to 0.9026 and ECE decreased to 0.0483, whereas near-mature F1 decreased to 0.4932; 44 samples were rejected. Selective prediction therefore improved reliability among retained samples but changed both coverage and class-specific performance. Temperature scaling was used as the default probability-calibration procedure in the subsequent end-to-end experiments, whereas the near-aware selective rule was retained only as a supplementary analysis of selective prediction.

### 3.5. End-to-End Evaluation on Original Cultivation Images

Because the intended application begins with raw cultivation images, end-to-end object-level performance was treated as the principal application-oriented evaluation. End-to-end testing was conducted on 370 original cultivation images containing 475 ground-truth *P. citrinopileatus* instances. Before candidate-box filtering, the complete pipeline achieved an object-level macro F1 of 0.7072 and a near-mature F1 of 0.4672. RF-DETR-Small generated 539 candidate instances. Of these, 461 were successfully matched to ground-truth instances, 14 ground-truth instances were missed, and 78 predictions remained unmatched (Table 6).

When classification performance was calculated only for successfully matched prediction–ground-truth pairs, the matched-instance macro F1 and near-mature F1 were 0.7735 and 0.5161, respectively. The lower object-level scores show that detection errors, including missed and unmatched predictions, further reduced maturity-recognition performance in the complete pipeline. Under the matching protocol, an unmatched prediction was a candidate box that could not be assigned to a ground-truth instance and was therefore counted as a false positive. This category can include duplicate detections, responses to non-target regions, and poorly localized boxes that failed to meet the matching criterion. A missed instance was a ground-truth fruiting body for which no predicted box satisfied the same criterion. The difference between the 78 unmatched predictions and 14 missed instances indicates that excess candidate generation, rather than failure to detect annotated instances, was the main detection-stage error in the unfiltered pipeline.

To assess the effect of false-positive suppression, candidate-box filtering was applied to the original detector output. Filtering reduced the number of predicted instances from 539 to 490 and the number of false-positive instances from 78 to 38, increasing detection precision from 0.8553 to 0.9224. Object-level macro F1 increased from 0.7072 to 0.7305, and object-level near-mature F1 increased from 0.4672 to 0.4885. Of the 49 predictions removed by filtering, 40 were unmatched predictions and nine had previously satisfied the matching criterion. Thus, 81.6% of the removed candidates were false positives, which accounts for the substantial increase in detection precision.

Candidate-box filtering also introduced a recall penalty. The number of matched instances decreased from 461 to 452, the number of missed instances increased from 14 to 23, and detection recall decreased from 0.9705 to 0.9516. The removal of nine previously matched predictions corresponded to the increase in nine missed ground-truth instances. Matched-instance macro F1 changed only slightly, from 0.7735 to 0.7752, indicating that the main effect of this post-processing step was to reduce unmatched predictions rather than alter classification performance for correctly localized fruiting bodies. Candidate-box filtering can therefore improve object-level performance when false positives are frequent, but this gain must be balanced against the corresponding loss in instance recall.

Together, RF-DETR-Small and the final morphology-guided ordinal classifier enabled complete inference from raw cultivation images to instance-level maturity outputs. The remaining gap between standalone classification and end-to-end performance indicates that, in addition to classifier errors, missed detections, false positives, and candidate-box quality at the detection stage materially affect object-level maturity recognition. The observed error pattern shows that candidate-box filtering primarily addressed excess detections, while stricter suppression converted a smaller number of previously matched instances into misses.

## 4. Discussion

This study developed a two-stage visual analysis pipeline for instance-level maturity recognition of *P. citrinopileatus* under controlled cultivation, combining instance detection, local-context dual-view representation, ordinal maturity modeling, and morphology-guided feature fusion. Unlike whole-image maturity assessment, the proposed approach first localizes individual fruiting bodies and then classifies their maturity stage, allowing fruiting bodies at different developmental stages to be handled within the same field of view. The framework is intended to provide spatially resolved maturity information for growth monitoring and harvest assistance rather than to replace harvest decision-making itself.

Deep learning has already been applied to mushroom identification, quality grading, growth monitoring, and harvest assistance. Previous research has noted the expanding use of computer vision and machine learning in the mushroom industry, while also identifying continuing limitations in scene adaptability, data standardization, and deployment-oriented evaluation [19]. For object localization, Mushroom-YOLO improved small-object detection of shiitake mushrooms by modifying YOLOv5 [20], whereas MYOLO optimized lightweight detection for fresh shiitake harvesting scenarios [21]. Further work combined shiitake fruiting-body detection with maturity, cap-texture, and harvesting-standard classification, illustrating the close relationship between localization and downstream state assessment [22]. Most previous studies have emphasized detection accuracy, lightweight deployment, or harvesting-grade classification, whereas the propagation of detection errors into downstream maturity recognition has received less attention. By evaluating detection and classification separately and then incorporating missed detections, false positives, and classification errors into an object-level analysis, the present study provides a clearer account of how errors at different stages affect the complete recognition pipeline.

RF-DETR-Small provided a favorable balance of localization performance under the present data conditions. In agricultural vision systems, a detector must not only identify targets but also provide bounding boxes suitable for downstream crop-based analysis. High recall alone does not necessarily make a detector the most suitable front end for a two-stage pipeline. In dense scenes with occlusion or closely adjacent boundaries, localization offsets and duplicate predictions can alter the content of subsequent instance crops and thereby affect maturity classification. RF-DETR-Small was selected on the validation set and used as the instance detector in the end-to-end pipeline. Conventional detector metrics and end-to-end object-level statistics serve different evaluation purposes and should therefore be interpreted separately: the former characterize standalone localization performance, whereas the latter reflect the practical quality of candidate instances entering the complete pipeline.

The local view focuses on direct maturity-related cues such as cap color, margin expansion, and overall morphology. The context view retains limited information about occlusion, neighboring fruiting bodies, and the local cultivation environment. Previous RGB-D-based research has shown that size, shape, and spatial structure can contribute to mushroom maturity assessment [23]. Because the present study used RGB images only, the context view cannot substitute for true depth information. Instead, it supplements local cues that may be lost in a tightly cropped target view. DINOv3 ViT-S/16 performed well for balanced accuracy and macro F1 under the validation-based model-selection protocol, indicating its suitability for fine-grained maturity discrimination in the present dataset.

The immature, near-mature, and mature classes have a clear ordinal relationship, while maturation itself is continuous. Modeling the three stages as fully independent classes disregards this rank structure. Ordinal methods such as CORAL and CORN exploit class order through rank-consistency or conditional-probability formulations [24,25]. The ordinal formulation used here explicitly incorporated relationships among adjacent maturity stages into instance-level visual modeling. Compared with the evaluated single-view approaches, the combined model achieved better QWK and MAE, indicating closer agreement between predictions and the ordered maturity structure. The near-mature class nevertheless remained the most difficult stage to recognize. This is consistent with its transitional nature: cap expansion, margin morphology, and color overlap with characteristics of both immature and mature fruiting bodies, while biological variation and uncertainty in manual stage assignment further blur the class boundary.

Morphological information was used as a complement to deep visual features rather than as a stand-alone rule-based classifier. Validation-set component comparisons showed that removing the morphology auxiliary loss improved macro F1, near-mature F1, QWK, and MAE. Accordingly, the final classifier did not retain this auxiliary supervision. The configuration retaining morphology input and feature fusion performed better than the variant in which the complete morphology pathway was removed. This comparison suggests that explicit morphology descriptors can provide useful information beyond the learned visual representation. Because the morphology input and fusion pathway changed together in some variants, this finding applies to the complete morphology-information pathway rather than to an isolated subcomponent.

Probability calibration provided an additional assessment of the reliability of the final classifier’s confidence estimates. The temperature parameter was estimated on the validation set and then fixed for the independent test set. Because temperature scaling did not alter the predicted labels, the principal classification metrics remained unchanged, whereas ECE decreased from 0.1094 to 0.0564. The uncalibrated probabilities therefore showed a measurable confidence gap that was reduced by temperature scaling. The near-aware selective decision rule further lowered ECE and increased the accuracy of retained samples but reduced coverage and near-mature F1. This result illustrates that improved reliability among high-confidence predictions does not necessarily produce better recognition for every maturity class. Temperature scaling is therefore appropriate when class predictions must remain unchanged, whereas selective prediction requires application-specific decisions concerning rejection rates and class-dependent risks.

Despite the overall classification performance, near-mature recognition remained the principal class-level limitation of the classifier. The near-mature F1 was 0.5546 in standalone ROI classification and decreased to 0.4672 in the end-to-end object-level evaluation. This difficulty may partly reflect the transitional morphology of near-mature fruiting bodies, which share cap, margin, color, and size characteristics with both adjacent stages. The lower representation of this class may also have contributed to its performance, because near-mature instances accounted for 11.1% of the classification dataset and only 53 test samples. Even after consensus review by the annotators, discrete labels cannot fully represent a continuous biological maturity boundary. The current system is therefore better suited to supporting harvest inspection than to independently determining harvest timing, particularly for low-confidence near-mature predictions.

In the end-to-end evaluation starting from raw cultivation images, the pipeline achieved an object-level macro F1 of 0.7072 and a near-mature F1 of 0.4672. These results provide a more application-oriented estimate than classification based on manually defined instance crops. The higher matched-instance macro F1 of 0.7735 indicates that practical performance depended on both maturity classification and detection quality. False-positive detections produced unnecessary maturity predictions, whereas missed detections excluded fruiting bodies from subsequent assessment. Inaccurate bounding boxes may also omit informative cap-margin regions or include neighboring fruiting bodies, thereby altering the local and context inputs. Candidate-box filtering reduced false positives and improved object-level F1, but it also increased the number of missed instances. Therefore, filtering thresholds should be selected according to the operational balance between false alarms and omitted fruiting bodies.

Although the independent test set was excluded from model selection, multiple architectural components and post-processing parameters were compared using the same validation set. Repeated use of one validation split may have favored configurations that performed particularly well under the present data partition. Moreover, the experiments used one original-image-level split and a fixed random seed. The reported comparisons therefore do not quantify variability arising from alternative data partitions or stochastic model training. Accordingly, the comparative results should be interpreted as evidence under the present experimental split rather than as estimates of performance variability across different partitions. Future studies should use repeated group-wise splits or cross-validation and report confidence intervals across independent training runs.

Several broader limitations should also be considered. The dataset was collected in a specific controlled-cultivation environment with a limited range of illumination, viewpoints, cultivation arrangements, and management conditions. Generalization across production sites, seasons, and growth batches therefore requires further evaluation. RGB images provide limited information about severe occlusion, overlapping growth, and three-dimensional structure. Depth, multi-view, or temporal observations may improve recognition under these conditions. The computational cost of RF-DETR-Small and DINOv3 ViT-S/16, together with long-term system stability, should also be evaluated under production conditions.

## 5. Conclusions

This study developed a two-stage framework that combines instance detection, local-context ordinal modeling, and morphology-guided feature fusion for individual maturity assessment of *P. citrinopileatus*. The results support the feasibility of producing instance-level maturity outputs directly from raw cultivation images, although end-to-end performance remained affected by false-positive detections, missed instances, bounding-box quality, and the limited recognition of near-mature fruiting bodies. These limitations indicate that the current framework is more appropriate for harvest assistance than for autonomous harvest decisions. Future work should evaluate the approach across production sites and repeated data partitions while incorporating temporal, depth, or multi-view information to improve near-mature recognition and cross-scene robustness.

## Figures and Tables

**Figure 1:**
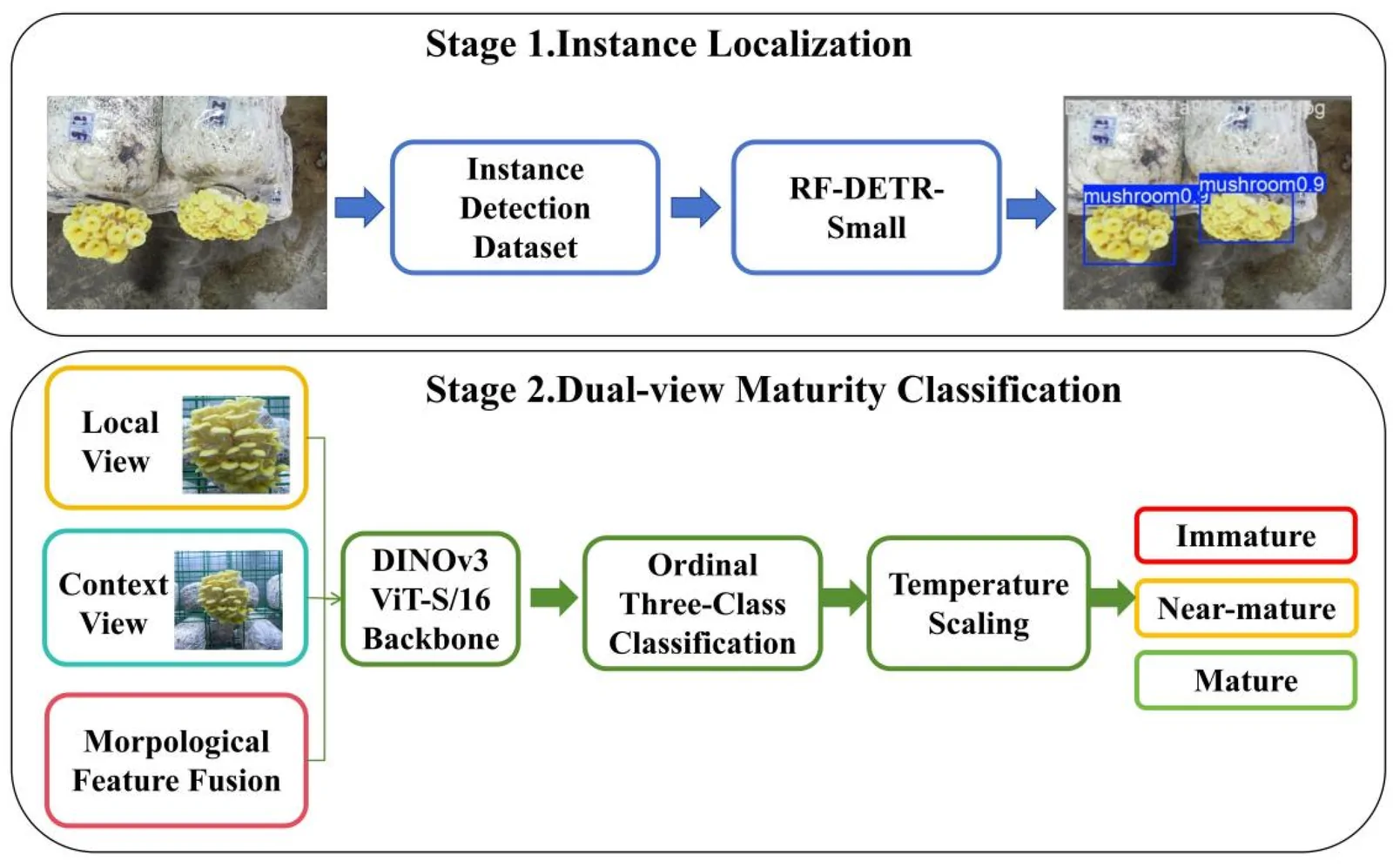
Overall workflow.

**Figure 2:**
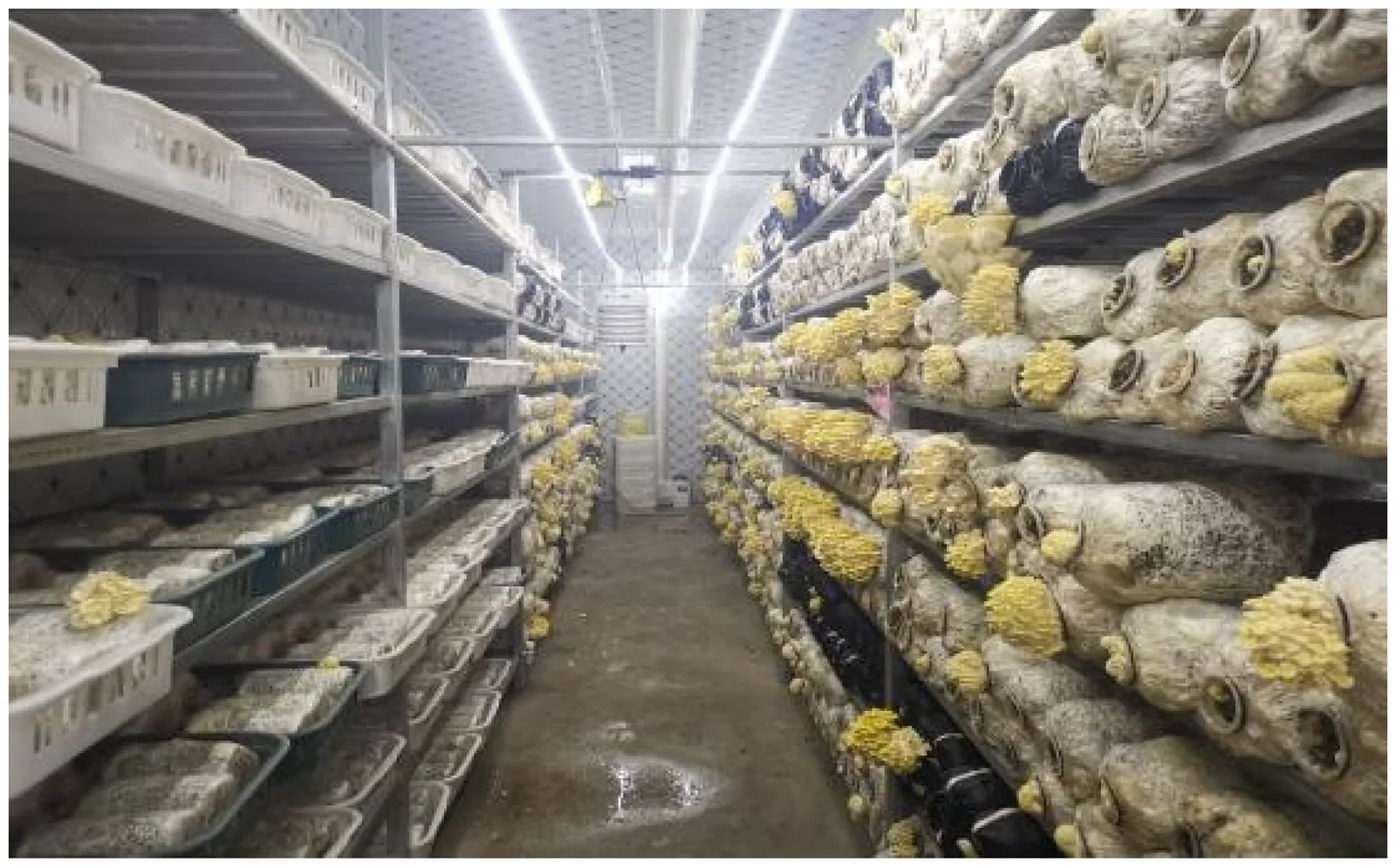
Experimental environment.

**Figure 3:**
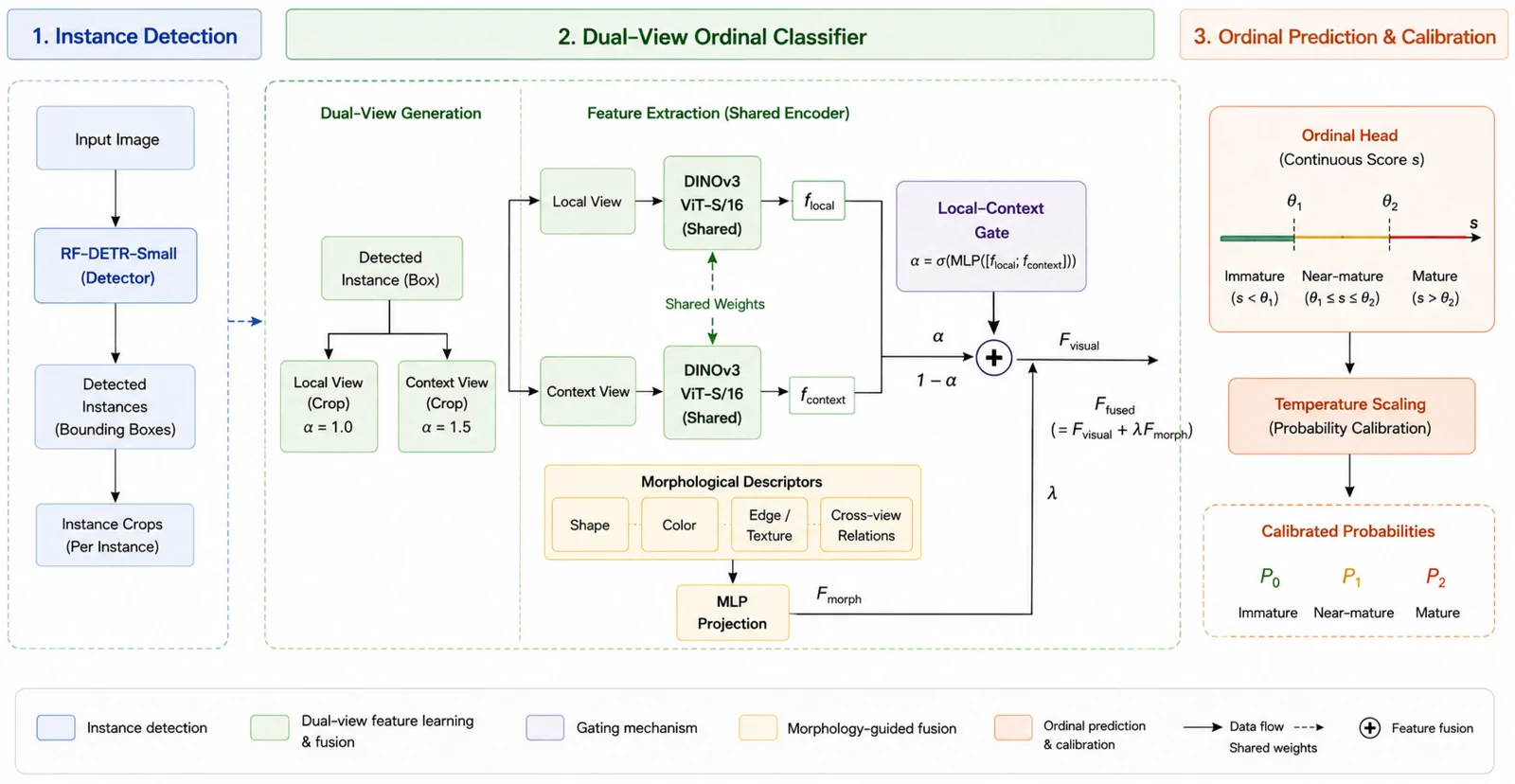
Architecture of the proposed morphology-guided ordinal classifier.

**Figure 4:**
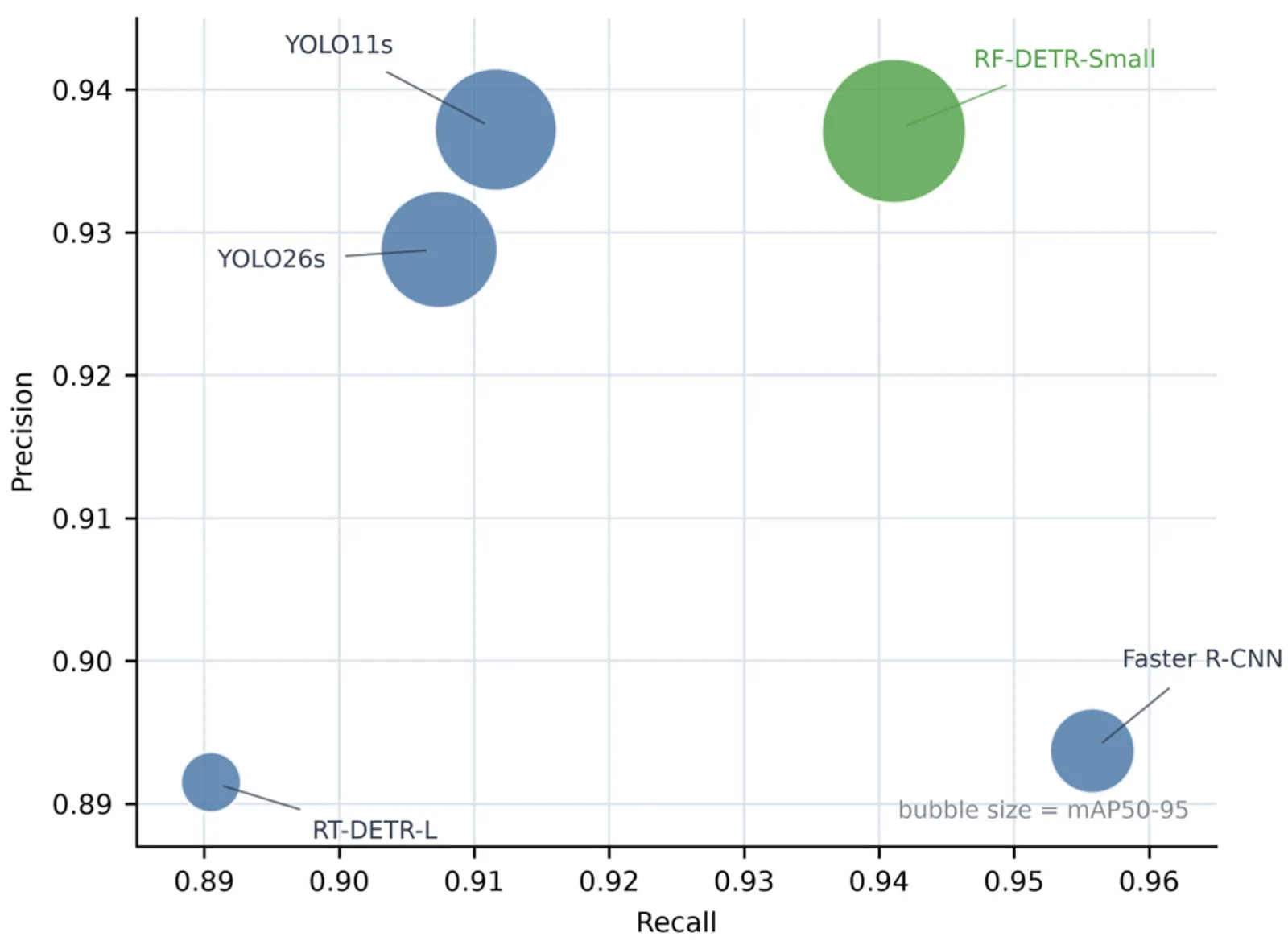
Performance trade-off among candidate instance detectors.

**Figure 5:**
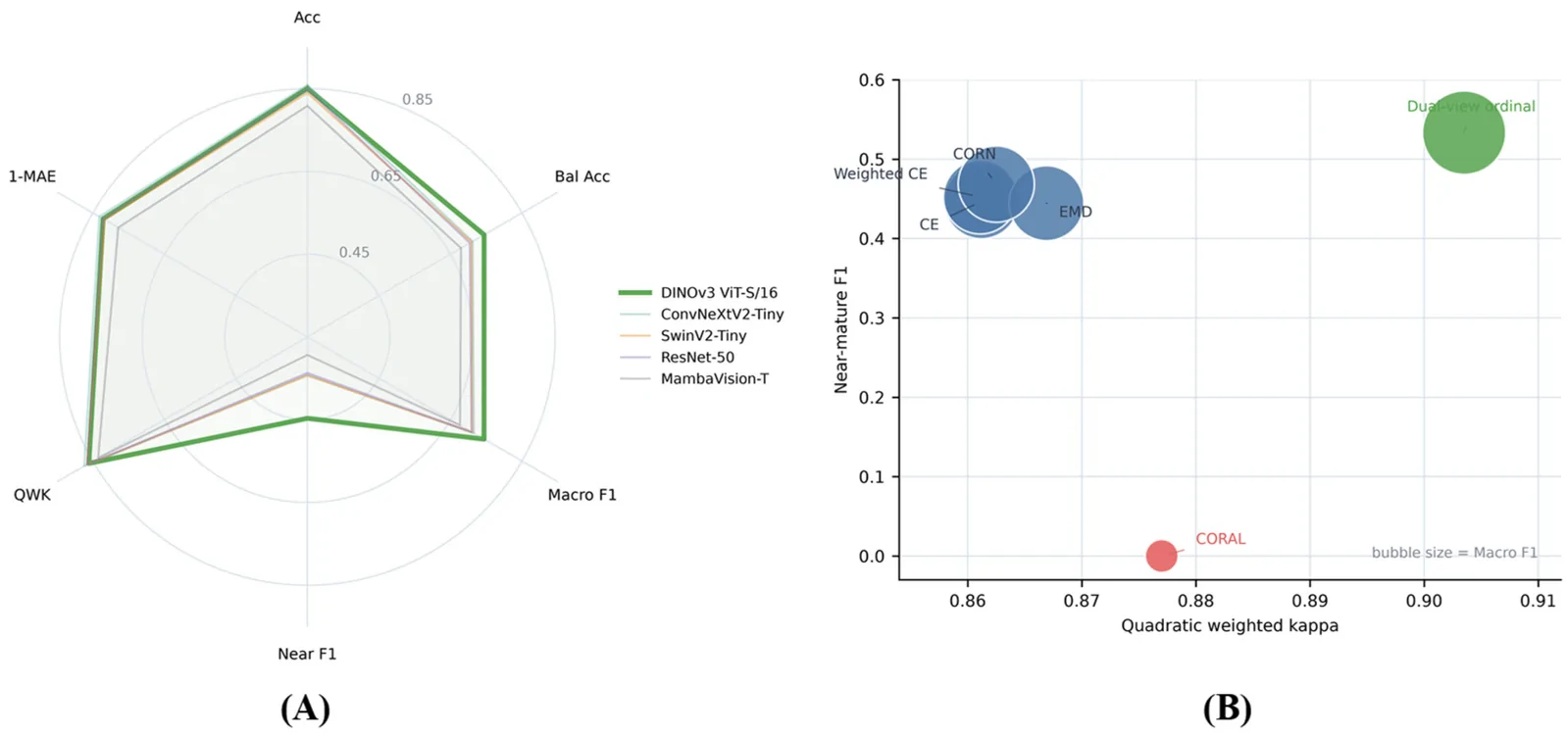
Backbone selection and maturity-modeling strategy comparison. (**A**) Backbone comparison. (**B**) Maturity-modeling strategy comparison.

**Table 1:** Detection performance of candidate instance detectors on the validation set.

Detector	mAP50	mAP50-95	Precision	Recall
RF-DETR-Small	0.9747	0.8813	0.9371	0.9411
YOLO11s	0.9751	0.8647	0.9372	0.9116
YOLO26s	0.9726	0.8612	0.9288	0.9074
Faster R-CNN R50-FPN V2	0.9538	0.8426	0.8937	0.9558
RT-DETR-L	0.9434	0.8323	0.8915	0.8905

**Table 2:** Backbone comparison under cross-entropy training on the validation set.

Backbone	Acc	Bal. Acc	Macro F1	Near F1	QWK	MAE
DINOv3 ViT-S/16	0.8508	0.7441	0.7439	0.4466	0.8612	0.1786
ConvNeXtV2-Tiny	0.8592	0.7119	0.7157	0.3415	0.8738	0.1681
SwinV2-Tiny	0.8403	0.7092	0.7111	0.3434	0.8659	0.1828
ResNet-50	0.8487	0.7043	0.7090	0.3371	0.8653	0.1786
MambaVision-T	0.8084	0.6795	0.6767	0.2936	0.8351	0.2211

**Table 3:** Comparison of maturity-modeling strategies based on DINOv3 ViT-S/16 on the validation set.

Setting	Acc.	Bal. Acc	Macro F1	Near F1	QWK	MAE
CE	0.8508	0.7441	0.7439	0.4466	0.8612	0.1786
Weighted CE	0.8421	0.7485	0.7408	0.4522	0.8611	0.1832
EMD	0.8484	0.7508	0.7457	0.4444	0.8669	0.1768
CORN	0.8484	0.7585	0.7517	0.4685	0.8625	0.1789
CORAL	0.8611	0.6438	0.6050	0.0000	0.8770	0.1663
Dual-view ordinal baseline	0.8716	0.7996	0.7873	0.5333	0.9035	0.1389

**Table 4:** Ablation study of the morphology-guided ordinal classifier on the validation set.

Variant	Gate	Rank	Morph. Input	Morph. Fusion	Morph. Aux	Acc	Macro F1	Near F1	QWK	MAE
Morphology-guided ordinal classifier + auxiliary loss	✓	✓	✓	✓	✓	0.8653	0.7661	0.4771	0.8951	0.1495
w/o morphology input	✓	✓	–	–	–	0.8632	0.7666	0.4828	0.8995	0.1474
w/o morphology fusion	✓	✓	✓	–	✓	0.8611	0.7740	0.5082	0.8941	0.1516
Final morphology-guided ordinal classifier	✓	✓	✓	✓	–	0.8737	0.7871	0.5299	0.9052	0.1368
w/o ranking loss	✓	–	✓	✓	✓	0.8653	0.7683	0.4821	0.8980	0.1474
w/o local-context gate	–	✓	✓	✓	✓	0.8674	0.7854	0.5484	0.8922	0.1474

✓ indicates that the corresponding component is included in the ablation experiment.

**Table 5:** Final classification performance and post-hoc probability calibration on the test set.

Rule	Acc	Bal. Acc	Macro F1	Near F1	QWK	MAE	ECE	Coverage	Rejected
Final classifier, raw argmax	0.8779	0.8053	0.7950	0.5546	0.9068	0.1326	0.1094	1.0000	0
Final classifier + temperature scaling	0.8779	0.8053	0.7950	0.5546	0.9068	0.1326	0.0564	1.0000	0
Near-aware retained set	0.9026	0.7827	0.7862	0.4932	0.9223	0.1090	0.0483	0.9074	44

**Table 6:** End-to-end object-level maturity recognition with and without candidate-box filtering.

Setting	Matched	FP	Det. Prec	Det. Rec	Obj. Macro F1	Obj. Near F1	Matched Macro F1	Matched Near F1
RF-DETR-S	461	78	0.8553	0.9705	0.7072	0.4672	0.7735	0.5161
RF-DETR-S-CBF	452	38	0.9224	0.9516	0.7305	0.4885	0.7752	0.5211

## Data Availability

The raw data supporting the conclusions of this article will be made available by the authors on request.

## Supplementary Materials

The following supporting information can be downloaded at: https://www.mdpi.com/article/10.3390/jof12100737/s1. Table S1. Prespecified paired comparisons of test-set Macro F1 across the ten matched random seeds. Table S2. Key paired comparisons of boundary Macro F1 across the ten matched random seeds.
