# Supplementary material for: Instance-Level Maturity Recognition of Pleurotus citrinopileatus via Ordinal Learning and Morphology-Guided Feature Fusion
Source: J Fungi (Basel). 2026 Oct 1;12(10):737. doi: 10.3390/jof12100737 (PMC13706209; doi:10.3390/jof12100737)
Supplement: Supplementary file 1 [file jof-12-00737-s001.zip › jof-4546707-supplementary.pdf]

Table S1. Prespecified paired comparisons of test-set Macro F1 across the ten matched random seeds.

Comparison	Mean difference	95% CI	Paired test	Holm-adjusted p	Cohen's dz
G4 vs G0	0.0238	0.0200 to 0.0276	t(9) = 14.22	< 0.001	4.50
G0+KD vs G0	0.0083	0.0035 to 0.0131	t(9) = 3.91	0.007	1.24
G0+soft label+KD vs G0+KD	0.0040	-0.0012 to 0.0092	t(9) = 1.73	0.119	0.55
RSG-BE+KD vs G0+KD	0.0126	0.0086 to 0.0166	t(9) = 7.20	< 0.001	2.28
G4 vs G0+soft label+KD	0.0115	0.0067 to 0.0163	t(9) = 5.48	0.001	1.73

TableS2. Key paired comparisons of boundary Macro F1 across the ten matched random seeds.

Comparison	Mean difference	95% CI	Paired test	Holm-adjusted p	Cohen's dz
G4 vs G0	0.0569	0.0472–0.0666	t(9) = 13.32	< 0.001	4.21
RSG-BE+KD vs G0+KD	0.0260	0.0158–0.0362	t(9) = 5.77	< 0.001	1.83
G4 vs G0+soft label+KD	0.0313	0.0226–0.0400	t(9) = 8.17	< 0.001	2.58
G4 vs RSG-BE+KD	0.0157	0.0075–0.0240	t(9) = 4.33	0.0019	1.37
